# Reliability and Validity of the Composite Activity-Related Fall Risk Scale

**DOI:** 10.3389/fneur.2022.832691

**Published:** 2022-03-22

**Authors:** Yan N. Jiang, Jing X. Wang, Lin Y. Chen, Jia J. Yao, Ling Ni, Jie M. Sheng, Xia Shen

**Affiliations:** ^1^Department of Rehabilitation Sciences, Tongji University School of Medicine, Shanghai, China; ^2^Department of Physical Therapy, Shanghai YangZhi Rehabilitation Hospital (Shanghai Sunshine Rehabilitation Center), School of Medicine, Tongji University, Shanghai, China; ^3^Rehabilitation Medicine Research Center, Shanghai YangZhi Rehabilitation Hospital (Shanghai Sunshine Rehabilitation Center), School of Medicine, Tongji University, Shanghai, China; ^4^Department of Rehabilitation Sciences, School of Medicine, Tongji University, Shanghai, China

**Keywords:** composite activity-related fall risk scale, psychometrics, older people, stroke, spinal cord injury

## Abstract

**Introduction:**

The newly developed Composite Activity-related Risk of Falls Scale (CARFS) is designed to measure composite activity-related risk of falls (CARF) in relation to the activity-specific fear of falling and physical behavior. This study tested the reliability and validity of the CARFS in older people with various health statuses and persons with stroke or spinal cord injury.

**Methods:**

Participants included 70 older adults, 38 persons with stroke, and 18 with spinal cord injury. They were first surveyed using a combined questionnaire including the CARFS and activity-specific balance confidence (ABC) scale in addition to items asking for personal and disease-related information, fall history, walking independence levels for examining internal consistency, ceiling and floor effects, and convergent validity in each participant group. One week after the initial survey, 33 older participants were reexamined using the CARFS to analyze test-retest reliability, where a minimal detectable change was found. Significance was set at α = 0.05 for all analyses.

**Results:**

The CARFS showed excellent test-retest reliability in the dimensions of fear of falling, physical behavior, and CARF [ICC (3,1) = 0.972, 0.994, and 0.994, respectively for their overall score], with a minimal detectable change of 3.944 in the older population. The internal consistency of CARFS items was excellent in the older participants, good in participants with stroke or spinal cord injury (Cronbach's alpha = 0.945, 0.843, 0.831 in each participant group, respectively). No ceiling and floor effects were demonstrated in the wide range of people. For the convergent validity, overall CARF score was significantly correlated with the average ABC score in each participant group (rho = −0.824, −0.761, and −0.601, respectively; *p* < 0.01), and was significantly correlated with walking independence levels in each participant group (rho = −0.636, −0.423, and −0.522, respectively; *p* < 0.01). It showed weak correlation with the number of previous falls only in participants with stroke (rho = 0.291, *p* = 0.076).

**Conclusion:**

The CARFS is a reliable and valid tool for measuring fall risk in older people and persons with stroke or spinal cord injury.

## Introduction

Falls are the most common cause of accidental injury among inpatients. They not only increase pain and financial burdens for these individuals, but may also result in medical disputes (1, 2). Nearly half of all falls cause physical injuries, with many severe cases leading to brain injury, internal organ damage, fractures, and even death (2). Related fear and anxiety can also create psychological damage, which may lead to dependency, thus increasing the burden of family care and severely impacting living quality (1, 3). This emphasizes the need for fall prevention both at the individual level and to ensure the integrity of health and social care services.

Risk assessment is crucial in fall prevention. Fear of falling, physical behavior, and physical functioning are psychosocial, lifestyle, and intrinsic risk factors of falls in elderly people, respectively (4), which intercorrelate with each other (5, 6). Fear of falling and physical behavior play dual roles in preventing falls, which are initially protective by making the person more aware of surroundings or avoiding exposure to activities that may lead to falling. However, this may be detrimental in the long term due to physical deconditioning induced (7, 8). The Composite Activity-specific Risk of Falls Scale (CARFS) was developed by Wang et al. to compositely evaluate the risk of falls by linking fear of falling and physical behaviors (9). It has questions on the degree of fear of falling (FoF) and performance frequency comprising 14 items of daily activities, and a score of Composite Activity-specific Risk of Falls (CARF) of each item calculated *via* a formula with FoF degree and performance frequency. To the best of our knowledge, the CARF is the first to consider and quantify dual influences of activity restriction through interactions with FoF on risk of falls (9). The Survey of Activities and FoF in the Elderly (SAFE) is an existing relevant assessment tool (10), which contains dimensions of FoF and activity restriction as well. However, Non-linkage between the two dimensions impedes the examination of dual influences of activity restriction and FoF on risk of falls. Besides, regarding activity restriction in the SAFE, subjects are asked to compare to 5 years ago to determine if and how restriction exists. Recalling 5-year memory makes the SAFE not suitable as an outcome measure of activity restriction in long-term evaluations or evaluations before and after interventions.

The CARFS is expected to be applicable for a wide range of people with different health statuses or different disability levels, since between-populations comparison in fall risks and long-term monitoring of fall risks are important to optimize resource and augment effectiveness in fall prevention. The Fall Efficacy Scale (FES) (11) or the Activities-specific Balance Confidence Scale (ABC) (12), the most common tool of activities-specific FoF assessment, have either ceiling effect for persons with better mobility (11), or floor effect for those with poor mobility (12). Thus, they are not suitable to use in between-populations comparison of fall risks or in long-term monitoring of fall risks for persons whose mobility changes largely. In development of the CARFS, respective interview responses from people with different health statuses (older persons, persons with stroke and persons with spinal cord injury) who had different disability levels was considered.

The CARFS has been approved with strong content validity by an expert panel (9). This study further assessed the reliability and validity of the CARFS in target populations. We hypothesized that the CARFS is reliable enough, has no ceiling and floor effects, correlates other measures on risk of falls, and applicable for different target populations.

## Materials and Methods

### Design

This study was designed to evaluate reliability and validity of the CARFS. Two questionnaire surveys were conducted with 1 week of rest in between. The first survey was performed in three target participant groups for examining internal consistency, checking ceiling and floor effects, and analyzing convergent validity (older adults, persons with stroke or with spinal cord injury). The second survey was performed only in the older participant group to explore test-retest reliability and calculate the minimal detectable change [MDC_(95)_] in the population. Older participants living in the community, and those with stroke or spinal cord injury who were admitted for at least a month when took part in the first survey were invited to complete the second survey, ensuring similar lifestyle components between surveys. The study was approved by the Ethics Committee of Shanghai YangZhi Rehabilitation Hospital affiliated with Tongji University (YZ2019-005).

### Participants

The participants consisted of three groups of individuals, particularly older persons over 60 years of age, persons with stroke, and persons with spinal cord injury. All participants were recruited from the Shanghai Yangzhi Rehabilitation Hospital affiliated with Tongji University and nearby resident communities using poster advertisements. For older participants, they were required to be aged 60 years and above and have adequate communication abilities to complete the survey. Individuals were excluded if they showed inadequate communication ability or had Mini-Mental State Examination scores of 23 or lesser (13). For participants with stroke or spinal cord injury, there was no criterion on age but rather on health status. For those who had suffered a stroke or spinal cord injury, other selection criteria were similar to those for the older participants in the first group. It must be noted that for older participants, no specific health status was required as a criterion. Older participants living in a community and those with stroke or spinal cord injury staying at a hospital were included into the older participant group in the evaluation of reliability and validity of the CARFS. All participants provided written informed consent prior to study engagement.

### Measures

This study implemented a general questionnaire asking for personal and health-related information, fall history, walking independence level, and balance confidence in addition to the CARFS. Personal information included gender, age, and education level, while health-related information included health status (healthy, stroke, spinal cord injury, or others) and time after disease onset. For falling history, participants were asked “Have you fallen within the past 6 months and how many times, if yes?” Here, a fall was defined as an event during which an individual came to rest on the ground or lower level, but not as the result of a major intrinsic event, such as a syncope, stroke, seizure, or overwhelming hazard (14). Walking independence levels were measured using the Functional Assessment Measure (FAM), including no disability (complete independence in a timely, safely manner), slight disability (modified independence with extra time or assistive devices), and severe disability (dependence with supervision or assistance) (15). Balance confidence was assessed using the ABC scale. It contains 16 items comprising different standing and walking activities. Participants rate their confidence in performing each activity without losing balance by selecting from values ranging from 0 (no confidence) to 100 (completely confident). Previous research has shown that the ABC has good psychometric properties for older people and patients with stroke (16, 17).

The CARFS contains 14 items and two activity-specific prompts on FoF and activity frequency, including “Think about the degree of FoF you feel when you perform the following activities” and “Think about how often you have performed the following activities over the last month”. A Likert scale ranging from 0 to 4 was used to quantify both FoF and activity frequency. For FoF, 0 indicates no worry at all, 1 indicates slight worry, 2 indicates moderate worry, 3 indicates high worry, and 4 indicates extreme worry. For activity frequency, 0 indicates none (have not done the activity over the last month), 1 indicates occasionally (within the last month), 2 indicates sometimes (weekly), 3 indicates often (daily), and 4 indicates very often (daily, at a higher frequency than normal). CARF scores were calculated based on the degree of FoF (A) and activity frequency (B) using the following formula: C = A + (4–B) + A ^*^ B/2, where 4-B reflects the restriction of activity (9). The CARF scores for each item ranged from 0 to 12 (9). The overall scores of the 3 dimensions including FOF, activity frequency, and CARF are calculated by the sum of each item score, which ranged from 0–56, 0–56, and 0–168, respectively. The full version of the CARFS is accessible in a previously published paper (9).

### Statistical Analysis

We conducted the statistical analyses using IBM SPSS version 21.0. First, we used descriptive statistics to describe all quantitative data. Subsequently, we analyzed test-retest reliability in the older participant group using intraclass correlation coefficient [ICC(3,1)] with two-way mixed model, single measure type (18). We further calculated the difference and mean of the overall CARF scores at the two assessments and employed Bland Altman plots to evaluate the degree of agreement between the test scores of the two assessments. Thereafter, with the ICC of the overall CARF score, we calculated the MDC_(95)_ through the formula: MDC_(95)_ = SEM^*^1.96^*^√2, where SEM = SDbaseline^*^√(1-ICC). The SDbaseline was the standard deviation of the overall CARF score at the first time. The %MDC_(95)_ was further calculated by the formula: %MDC = MDC_(95)_/168 ×100% (18).

Afterwards, we evaluated internal consistency of the CARFS items using Cronbach's alpha in each participant group. Subsequently, we checked the ceiling and floor effects through the frequency plot of the overall CARF score. Finally, for examining convergent validity, we used Spearman's correlation to explore the correlation of the CARFS with the ABC score, and independence level of walking measured by FAM, and number of previous falls.

We classified ICC and Cronbach's alpha values as poor (<0.50), moderate (0.50–0.75), good (0.75–0.90), and excellent (≥0.90) (19). For floor and ceiling effects, we set the proportion of the highest or lowest CARF score higher than 15% of target participants (20). For the correlation between the CARFS and other fall risk measures, we graded the rho values as very weak (<0.20), weak (0.20–0.39), moderate (0.40–0.59), strong (0.60–0.79), and very strong (0.80–1.00) (21). Significance was determined at *p* ≤ 0.05. All *p* values were 2-tailed.

## Results

### General Participant Characteristics

The first survey comprised 98 participants, including 70 older adults aged 60 years, 38 adults with stroke, and 18 adults with spinal cord injury.

Among older participants, there were 42 common older persons without neurological disorders, 22 with stroke and six with spinal cord injury. They showed a more equal sex ratio than persons with stroke or spinal cord injury. The mean disease duration was 7.6 ± 6.7 months for participants with stroke, and 10.9 ± 5.8 months for those with spinal cord injury.

The second survey comprised 33 older adults including 29 older persons living at the community without any neurological disorder, two having previously suffered a stroke, and two with spinal cord injuries who had been in the hospital for over 1 month during the first survey. Another 13 common older persons without neurological disorders failed to complete the second survey because they had no time or lost connection during the second week of the survey.

Detailed participant characteristics are shown in Table 1.

**Table 1:** Participant characteristics.

**Characteristics**	**Older (*n* = 70)**	**Stroke (*n* = 38)**	**SCI (*n* = 18)**	**Older (*n* = 33)^#^**
Sex (Male:Female)	35:35	27:11	13:5	14:19
Age (year)^∧^	68.0 ± 5.4	55.8 ± 18.3	48.4 ± 15.7	67.7 ± 5.6
Education (median)	Secondary	Higher	Secondary	Secondary
Health status				
Healthy w/o motor impairment	42	–	–	29
Hemiplegia (duration, month)^∧^	22 (7.9 ± 7.3 m)	38 (7.6 ± 6.7 m)	–	2
Paraplegia (duration, month)^∧^	3 (12.3 ± 2.3 m)	–	10 (9.0 ± 4.9 m)	1
Quadriplegia (duration, month)	3 (10.7 ± 6.7 m)	–	8 (13.0 ± 6.4 m)	1
Walking ability				
Complete independence	52	21	1	29
Modified independence	2	2	2	1
Dependence	16	15	15	3
Number of previous falls				
0	62	33	13	30
1	7	5	3	2
2	1	0	2	1
Average ABC^∧^	81.9 ± 23.9	71.4 ± 19.9	27.5 ± 18.6	88.9 ± 21.8
Overall CARF score^@^	23.8(3.0–199.0) (3–119)	34.3(19.0–72.5)	89.3(53.5–121.5)	19.0(3.0–119.0)

# *Older participants who completed two surveys;*

∧ *Data are presented with mean and standard deviation;*

@ *data are presented with median and range*.

*SCI, spinal cord injury*.

### Test-Retest Reliability and Minimal Dateable Change

The three dimensions of the CARFS showed good to excellent repeatability for all items (ICC = 0.766–1.000) except for FoF of walking on wet ground which showed moderate reliability (ICC = 0.655). The ICC of the overall CARF score was 0.994, indicating excellent test-retest reliability. The results are shown in Table 2.

**Table 2:** Test-retest reliability of the CARFS.

**Items**	**ICC (95% CI)**		
	**FOF**	**Frequency**	**CARF score**
1. Sitting down & standing up	0.943 (0.889–0.972)	1.000 (1.000–1.000)	0.857 (0.731–0.927)
2. Bending down & straightening up	0.986 (0.972–0.993)	1.000 (1.000–1.000)	0.978 (0.956–0.989)
3. Standing activities	1.000 (1.000–1.000)	1.000 (1.000–1.000)	1.000 (1.000–1.000)
4. Squatting activities	0.974 (0.947–0.987)	0.987 (0.973–0.993)	0.977 (0.955–0.989)
5. Transferring while sitting	1.000 (1.000–1.000)	1.000 (1.000–1.000)	1.000 (1.000–1.000)
6. Walking short distances	1.000 (1.000–1.000)	1.000 (1.000–1.000)	1.000 (1.000–1.000)
7. Walking long distances	1.000 (1.000–1.000)	0.888 (0.785–0.943)	0.965 (0.931–0.983)
8. Walking on wet ground	0.655 (0.406–0.813)	0.982 (0.964–0.991)	0.766 (0.578–0.877)
9. Walking on uneven ground	0.915 (0.836–0.957)	1.000 (1.000–1.000)	0.876 (0.764–0.937)
10. Using transportation	0.972 (0.944–0.986)	0.961 (0.922–0.980)	0.959 (0.918–0.979)
11. Washing oneself	0.980 (0.961–0.990)	0.808 (0.647–0.901)	0.958 (0.916–0.979)
12. Toileting	0.982 (0.964–0.991)	1.000 (1.000–1.000)	0.975 (0.951–0.988)
13. Putting on/taking off trousers	1.000 (1.000–1.000)	1.000 (1.000–1.000)	1.000 (1.000–1.000)
14. Putting on/taking off footwear	1.000 (1.000–1.000)	1.000 (1.000–1.000)	1.000 (1.000–1.000)
Overall	0.972 (0.953–0.983)	0.994 (0.991–0.997)	0.994 (0.988–0.997)

*ICC, Intraclass Correlation Coefficients*.

**Figure 1:**
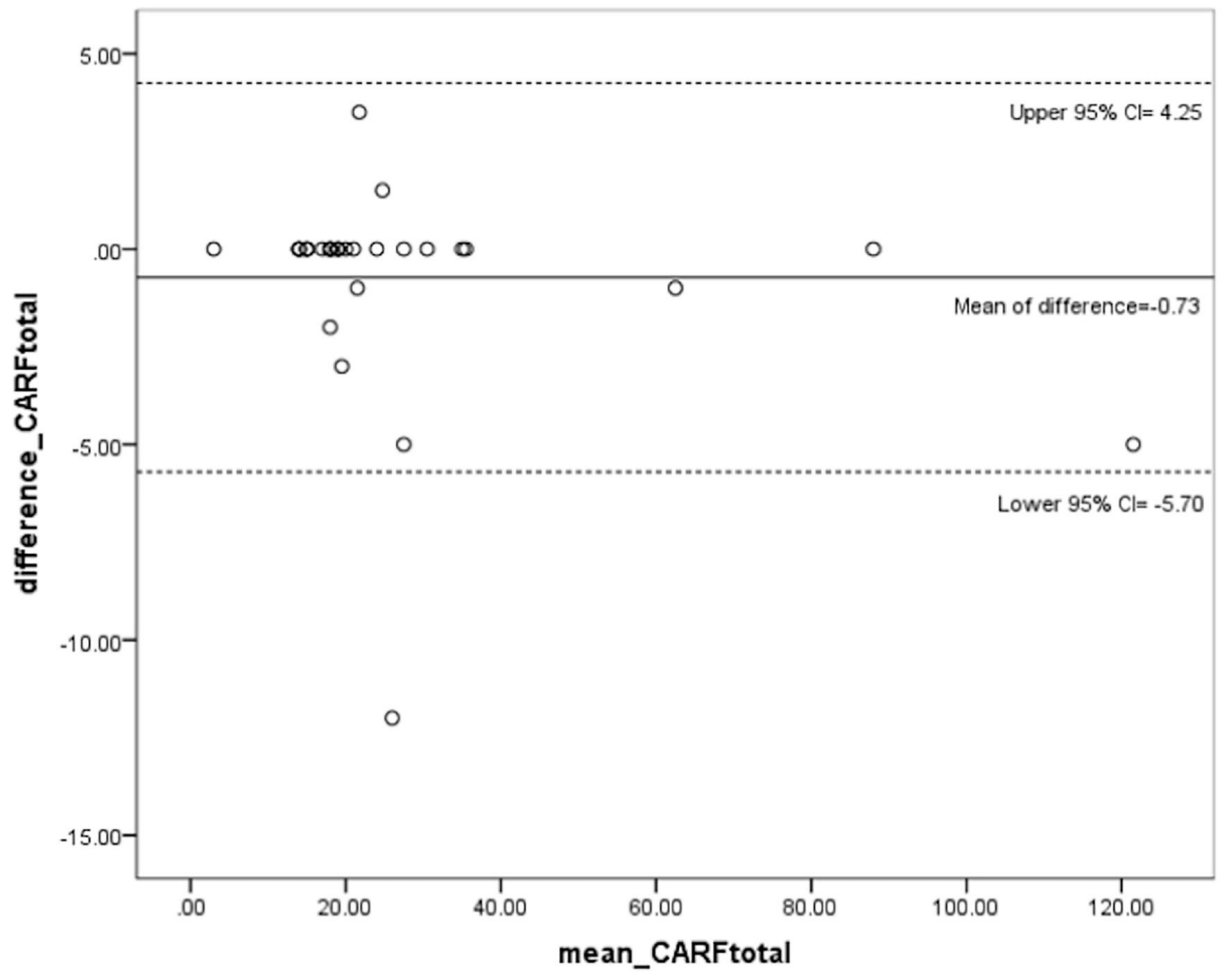
The Bland Altman plot of the overall CARF score between two assessments of test-retest.

The mean difference of the overall CARF scores at the two assessments was −0.7 (95% CI: −5.70 to 4.25). From the Bland Altman plot, only one extreme change exceeded the 95% CI. The result implies excellent repeatability for the overall CARF score. For the extreme change, it occurred in an older person without a neurological disorder, and arose from the change of two items, that is, walking on wet ground and walking on uneven ground. The Bland Altman plot is shown in Figure 1.

Based on the ICC value of 0.994, the MDC_(95)_ was calculated as 3.944 [% MDC_(95)_: 2.35%].

### Internal Consistency

For older participants, Cronbach's alpha for CARFS items was 0.945. A stepwise deletion of each of the 14 items did not alter the internal consistency for the CARFS (Cronbach's alpha if item deleted: 0.938–0.946). The item-total correlation was moderate to very strong, (coefficient: 0.523–0.850) for all items. The result indicated excellent internal consistency of the CARFS items in the elderly participants (Table 3).

**Table 3:** Internal consistency of the CARFS in the elderly, persons with stroke or with spinal cord injury respectively.

**Items**	**Cronbach's alpha if**			**Corrected item-total**			**Cronbach's alpha**		
	**item deleted**			**correlation**					
	**Older**	**Stroke**	**SCI**	**Elderly**	**Stroke**	**SCI**	**Elderly**	**Stroke**	**SCI**
1. Sitting down & standing up	0.941	0.836	0.819	0.698	0.441	0.485	0.945	0.843	0.831
2. Bending down & straightening up	0.940	0.825	0.807	0.754	0.603	0.674			
3. Standing activities	0.945	0.845	0.837	0.590	0.300	0.216			
4. Squatting activities	0.939	0.833	0.822	0.802	0.496	0.482			
5. Transferring while sitting	0.943	0.839	0.806	0.624	0.387	0.634			
6. Walking short distances	0.946	0.850	0.838	0.523	0.166	0.161			
7. Walking long distances	0.939	0.841	0.835	0.793	0.353	0.253			
8. Walking on wet ground	0.942	0.830	0.835	0.694	0.543	0.132			
9. Walking on uneven ground	0.940	0.836	0.841	0.754	0.438	−0.042			
10. Using transportation	0.938	0.825	0.823	0.850	0.687	0.439			
11. Washing oneself	0.939	0.827	0.796	0.780	0.571	0.762			
12. Toileting	0.938	0.821	0.805	0.809	0.653	0.659			
13. Putting on/taking off trousers	0.941	0.829	0.799	0.741	0.557	0.712			
14. Putting on/taking off footwear	0.941	0.823	0.797	0.722	0.622	0.726			

*SCI, spinal cord injury*.

In patient groups, the CARFS items showed good internal consistency with Cronbach's alpha of 0.843 and 0.831 in the stroke group and spinal cord injury group, respectively. The internal consistency for the CARFS did not change much if any item was deleted in both patient groups. (Cronbach's alpha if item deleted: 0.821–0.850, and 0.797–0.841 in the stroke group and spinal cord injury group, respectively). The item-total correlation was moderate to strong for most items (coefficient: 0.438–0.687), weak for three items (coefficient: 0.300–0.387), and very weak for one item (coefficient: 0.166) in the stroke group. It was moderate to strong for 9 items (coefficient: 0.439–0.726), weak for two items (coefficient: 0.216–0.253), very weak for three items (coefficient: −0.042–0.161) in the spinal cord injury group (Table 3).

### Ceiling and Floor Effects

**Figure 2:**
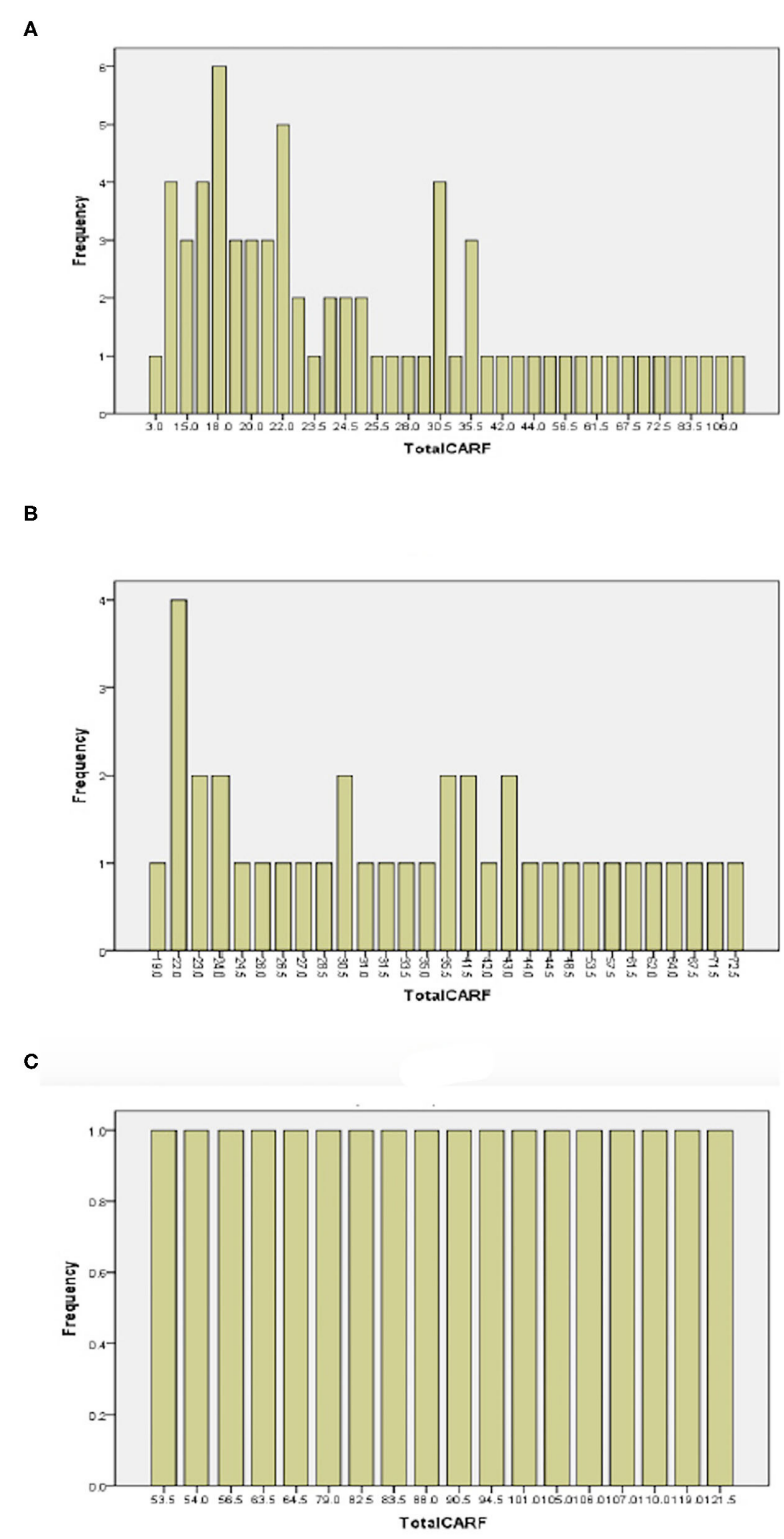
Frequency plots of overall CARF score in older participants **(A)**, persons with stroke **(B)**, and persons with spinal cord injury **(C)**.

More than 85% of older persons scored the CARF between 17.0–119.0. All persons with stroke scored between 19.0–72.5 and all persons with spinal cord injury scored between 53.5 and 12.5. The results indicate no ceiling or floor effects observed in any target participant group (Figure 2).

### Convergent Validity

The overall CARF score was strongly to very strongly correlated with the average ABC score in each participant group (rho = −0.824, −0.761, and −0.601, respectively; *p* < 0.01), and was moderately to strongly correlated with the walking independence levels in each group (rho = −0.636, −0.423, and −0.522, respectively; *p* < 0.01). It showed weak correlation with number of previous falls only in the group with stroke (rho = 0.291, *p* = 0.076). The average ABC score showed weak to moderate correlation with the walking independence levels in each group [rho = 0.603, 0.331, and 0.325 for elderly (*p* < 0.01), stroke (*p* < 0.05), and spinal cord injury groups, respectively (*p* > 0.05)]. The average ABC showed moderate correlation with number of previous falls only in the group with stroke (rho = 0.430, *p* < 0.05) (Table 4).

**Table 4:** Convergent validity of the CARFS on relation with other fall risk measures.

	**CARFS**				
	**Overall CARF score** **(0–168)**	**Overall FOF score** **(0–56)**	**Overall performance** **frequency (0–56)**	**ABC (0–100)**	**Walking independence** **level (1/2/3)^#^**
**Elderly (*N* = 70)**					
ABC (0–100)	−0.824^**^	−0.856^**^	0.679^**^		
Walking independence level (1/2/3)	−0.636^**^	−0.614^**^	0.700^**^	0.603^**^	
No of falls	0.197	0.224^!^	0.067	−0.181	0.003
**Stroke (*N* = 38)**					
ABC (0–100)	−0.761^**^	−0.811^**^	0.456^**^		
Walking independence level (1/2/3)	−0.423^**^	−0.356^**^	0.480^**^	0.331^*^	
No of falls	0.291^!^	0.288^!^	0.104	−0.430^*^	−0.142
**SCI (*N* = 18)**					
ABC (0–100)	−0.601^**^	−0.694^**^	0.604^**^		
Walking independence level (1/2/3)	−0.522^**^	−0.532^**^	0.573^**^	0.325	
No of falls	0.039	0.154	−0.424^!^	−0.155	0.002

** *P < 0.01*.

* *p < 0.05*.

! *p < 0.10*.

# *Walking independence level:1: Dependence, 2: Modified independence, 3: Complete independence*.

*SCI, spinal cord injury*.

## Discussion

The purpose of this study was to evaluate the reliability and validity of the newly developed CARFS in various target populations. Our results provide preliminary evidence for its reliability and validity in the assessment of fall risk among older persons, persons with stroke or with spinal cord injuries.

### Reliability

The ICC values for overall CARF score, FoF degree, and performance frequency rank were 0.994, 0.972, and 0.994, respectively, all denoting excellent reliability. Most ICC values for the three dimensions of each activity item were over 0.700 indicating good to excellent reliability except for one FoF score of walking on wet ground that was 0.655 implying moderate reliability. Powell et al. have reported the exceptional items of ABC scale with poor reliability as well (test-retest *r* < 0.40, car transfer and walking at home), in spite of excellent reliability for the overall ABC score (*r* > 0.90) (12). Although exact reasons of the exceptions were difficult to track, for questionnaires on FoF like ABC and FES, making hypothetical responses for activities which subjects have not experienced for a long time or have restricted totally, is a common manner which may lead to inaccurate FoF score and thereby affect test-retest reliability (11, 12). The data of activity frequency, although by recalling memories, could be more accurate than the psychological estimation of FoF. The concept is supported by our results that the activity frequency demonstrated higher test-retest reliability than FoF in most items of CARFS. Linking FoF with activity frequency, the CARF scores showed good to excellent reliability in all items. Observing the Bland Altman plot of difference of the overall CARF scores at the two assessments, the difference value of all persons located within the 95% CI except for one person's data. Thus, we can conclude that the CARFS has good to excellent test-retest reliability.

The MDC(95) of the CARF was 3.944, which implied that 95% of older adults showed random variation of fewer than 3.944 points in the CARFS. Thus, when the CARFS is adopted to monitor fall risk change for a certain period, a change of 3.944 or more is considered to be a true change. The % MDC of the CARFS was 2.35%, which is much lower than that of common survey tools, such as ABC scale (13%), Berg balance scale (9%), and 36-Item Short Form Survey (28%) used in people with Parkinson's disease (22). Lower % MDC could indicate greater competence to detect the change of fall risk in target population.

The Cronbach's alpha of the CARFS used in older adults was 0.945, implying excellent internal consistency of the CARFS. The item-total correlation of each CARFS items ranged from 0.523 to 0.850, which is superior than the result found in the ABC and SAFE scales used with older persons (10, 12). However, the Cronbach's alpha of the CARFS in patient groups was lower than that in older participants. Smaller sample size in patient groups than the older group could be an important factor contributing to the result. Because based on the formula of Cronbach's alpha, larger sample size produces larger Cronbach's alpha if other variables are kept the same (23). Generally, the value of 0.843 and 0.831 of Cronbach's alpha can still indicate good internal consistency of the CARFS in participants with stroke or with spinal cord injury.

### Validity

The overall CARF score ranged from 3.0–119.0 in older persons, from 19.0 to 72.5 in persons with stroke, and from 53.5 to 121.5 in persons with spinal cord injury. The maximum range of overall CARF score is 0–168. Therefore, ceiling effects did not occur in all participants. Observing the frequency chart, more than 85% of older persons scored over 17.0, indicating no floor effect in older persons, as well as in persons with stroke or spinal cord injury. Hence, we can conclude that there is no ceiling and floor effects in various ranges of population, including the older population, and people with either stroke or spinal cord injury.

For convergent validity, the overall CARF scores had a strong to very strong correlation with the ABC score (rho = −0.824, −0.761, and −0.601, respectively; *p* < 0.01) and had a moderate to strong correlation with the walking independence level in each participant group (rho = −0.824, −0.761, and −0.601, respectively; *p* < 0.01) (rho = −0.636, −0.423, and −0.522, respectively; *p* < 0.01). However, only a weak correlation was found with the number of previous falls in stroke participants (rho = 0.291, p = 0.076). The ABC scale has been found sensitive to discriminate individuals who are likely to suffer a fall in the elderly population with a cut-off value of 67 (24). In our study, the ABC scale had a weak, Non-significant correlation with the number of previous falls in older participants. We noticed that the rate of falling was only 11% in older participants, which is much lower than 36%, as found in previous studies (24, 25). Additionally, in our study, only one participant had recurrent falls in the previous 6 months, much <40% of recurrent fallers rate reported by the World Health Organization (26). Inadequate representativeness regarding falls features of our sample could be an important factor resulting in both ABC and CARFS providing a Non-significant correlation with the number of falls. The same situation about falls characteristics existed in the samples of participants with stroke or spinal cord injury. In stroke participants, although lacking representativeness, the CARFS showed near-to-significant weak correlation with the number of falls (rho = 0.291, *p* < 0.1), whilst the ABC had higher correlation with the number of falls (rho = −0.430, *p* < 0.1). The validity of the CARFS on correlating with fall history needs further examination in representative samples. The recruitment strategy should be modified to include greater frail elderly individuals, such as patients from nursing homes. Generally, CARFS showed moderate to strong convergent validity on correlating with the psychological and physical intrinsic risk factors of falls.

This study produced evidence suggesting that the CARFS is reliable and valid for use among populations with different health statuses. However, there were some limitations as well. First, although the sample size was much larger than that implemented in the pilot study (9), the representativeness of the target population is still not sufficient in terms of the demographic features and falls characteristics. Thus, a larger sample size is needed to improve representativeness. Second, this study did not explore a series of psychometric properties within the CARFS, including predictive validity to falls, and sensitivity to change. Additional research is needed to investigate these elements. Third, the CARFS is expected to be useful to provide guidance on designing fall prevention programs based on dual effects of activity restrictions on fall risk reflected in the CARF score. Hence, further studies are needed to assess the applicability and effectiveness of recommended fall prevention programs.

In conclusion, the CARFS is a reliable and valid tool for quantifying the composite activity-specific risk of falls in older people and persons with stroke or spinal cord injury. Future studies with representative samples are needed to explore the predictive validity of falls, sensitivity to change, and to testify applicability and effectiveness of guiding fall prevention in different target populations.

## Data Availability Statement

The original contributions presented in the study are included in the article/supplementary material, further inquiries can be directed to the corresponding author.

## Ethics Statement

The studies involving human participants were reviewed and approved by Ethics Committee of Yangzhi Affiliated Rehabilitation Hospital of Tongji University (YZ2019–005). The patients/participants provided their written informed consent to participate in this study.

## Author Contributions

XS conceived and designed the study. YJ, JW, LC, JY, LN, and JS performed data acquisition and analysis. YJ interpreted the data and drafted the manuscript. All authors read, revised the paper, and approved the final manuscript.

## Funding

This study was supported by the Shanghai Disabled Persons Federation Research Project (K2018029) and a research project of the Shanghai YangZhi Rehabilitation Hospital (HXHZ-015).

## Conflict of Interest

The authors declare that the research was conducted in the absence of any commercial or financial relationships that could be construed as a potential conflict of interest.

## Publisher's Note

All claims expressed in this article are solely those of the authors and do not necessarily represent those of their affiliated organizations, or those of the publisher, the editors and the reviewers. Any product that may be evaluated in this article, or claim that may be made by its manufacturer, is not guaranteed or endorsed by the publisher.
